# Virtual health care center in Georgia

**DOI:** 10.1186/1746-1596-3-S1-S4

**Published:** 2008-07-15

**Authors:** Thomas Schrader, Ekaterina Kldiashvili

**Affiliations:** 1 Department of Pathology, University Hospital Berlin – Charite. 10117-Berlin, Germany; 2 Georgian Telemedicine Union (Association). 0171-Tbilisi, Georgia

## Abstract

Application of telemedicine systems to cover distant geographical areas has increased recently. However, the potential usefulness of similar systems for creation of national networks does not seem to be widely appreciated. The article describes the "Virtual Health Care Knowledge Center in Georgia" project. Its aim was the set up of an online integrated web-based platform to provide remote medical consultations and eLearning cycles. The project "Virtual Health Care Knowledge Center in Georgia" was the NATO Networking Infrastructure Grant dedicated for development of telemedicine in non-NATO countries. The project implemented a pilot to organize the creation of national eHealth network in Georgia and to promote the use of innovative telemedicine and eLearning services in the Georgian healthcare system. In June 2007 it was continued under the NATO Networking Infrastructure Grant "ePathology – Virtual Pathology Center in Georgia as the Continuation of Virtual Health Care Center".

## Introduction

Telemedicine involves providing health care services between two or more locations through the use of telecommunication technology. A number of medical services including radiology, pathology as well as consultations in specialized disciplines like neurology, dermatology and cardiology can readily be delivered or accessed remotely using information technology [1]. Telepathology is a part of telemedicine, which can be defined as the practice of pathology over a distance by viewing images transmitted from a remote site [2,3]. The former classification of telepathology differentiates between the static and dynamic mode. In the static mode, a small number of digitized images are captured and transmitted to a remote telepathologist. In the dynamic mode, images of the microscopic slides are transmitted and visualized in real time. Some of the dynamic systems allow the robotic remote control of the microscope. Hybrid telepathology systems combine features of both static-image and dynamic-robotic telepathology systems. The current development of virtual microscopy using whole slide images (WSI) allows the static mode as well as the dynamic mode.

The images may be used for primary diagnosis, consultation, quality assurance, and proficiency testing and distance education. Telepathology has the potential to increase access to general or subspecialty pathology service in remote areas, decrease costs by reducing turn around time for consultations, and reduce professional isolation of the rural pathologist [4].

In this article NATO Networking Infrastructure Grants "Virtual Health Care Knowledge Center in Georgia" and "ePathology – Virtual Pathology Center in Georgia as the Continuation of Virtual Health Care Center" as well as barriers and perspectives of telepathology practice are presented.

## Methods and discussion

### Description

The project "Virtual Health Care Knowledge Center in Georgia" started at March 2005. Its topic was the implementation, evaluation and adaptation of telemedicine services for Georgia. The first step of this project implementation which aimed set up and installation of the teleconsultation server realized by NATO-country project co-director, Dr. Thomas Schrader has been completed at June 2005. The service can be reached at . Three main services are available on the server: eGroupWare (GroupWare), Moodle (Online Course Management System/LMS), Simple Machines Forum (TeleConsulting). eGroupWare is a type of software that allows users to share documents, calendar and addresses, plan projects or manage news. The main aspect for choosing it is large community and the fact that Virtual Health Care Knowledge Center in Georgia (VHCC) is "young" project with a promising future. Within the scope of telemedicine, teleconsulting is an important sub area, where several cases can be discussed with experts all over the world. The main idea for TeleConsulting tool was the possibility to create a case by an external team. The essential data are stored on the server and they are viewable by all interested groups. The last one can comment the questions and requests. After finalization the case is stored on the server and can be viewed by the users. Simple Machines Forum (SMF) is used for teleconsulting. A learning management system (LMS) is a software application of web-based technology used to plan, implement and access a specific learning process. There are several LMSs. The Moodle was determined as the most suitable and appropriate tool.

The usage of VHCC's teleconsultation server started at July 2005. The medical discussion group cared more than 140 cases. Other groups are specialized in Medical Informatics and Pathology. More than 250 pupils use the eLearning Platform of the VHCC with 15 different courses and about 25 teachers. The VHCC represents a living community with a high activity. Second opinion consultations at TeleConsulting are implemented on English and on Georgian. eLearning platform is realized on English, Georgian and German languages. For videoconferencing the Skype-tool was used.

In June, 2007 the project "ePathology – Virtual Pathology Center in Georgia as the Continuation of Virtual Health Care Center in Georgia" started. The main goal is to expand the infrastructure of Virtual Health Care Center (VHCC) in Georgia and to develop a new model to implement and promote second opinion consultations and education in the medical field of pathology (telepathology). The project proposes to create a unified, resilient and transparent infrastructure, available on demand, in order to expedite pathology consultants between rural pathology laboratories and specialized medical centers. Special emphasize is placed on: (1) distribution of computational resources; (2) development of image processing algorithms; (3) combination of image data with patient's medical data; (4) interoperability of databases of heterogeneous content for medical and research purposes. This model should assure a timely and secure access of medical data, as well as combine a wide variety of distributing resources, which should work under a single and unified environment.

It should be especially described, that VHCC project's patient-record is based on "events", i.e., episodes that occur during the patient's contacts with the healthcare organization. The patient's record is the collection of all his/her events; these medical data could be heterogeneous, ranging from numerical values to radiographical images. A relevant step forward is that information is gathered during routine patient treatment, not during activities explicitly dedicated to scientific research within Universities or research Institutes. Educational module slides will be already collected in a central database.

To ensure the implementation of VHCC in the most effective degree the content management system will be created. This will be the virtual model of diagnostical center, which will realize the concept of digital medical history.

### Barriers

The probability of an incorrect handling of a relevant medical data, still dangerously high, mainly is due to:

• Environmental factors – Many medical organizations are not fully able to face every disease, e.g., in a peripheral hospital only the most frequent pathologies for that geographical area are treated.

• Instinctive factors – The decision making of a physician is usually mainly based on the limited number of cases in her/his experience and/or on a static medical knowledge available from databases of main published slides. This factor is very variable between different specialists and general practitioners.

• Emotional factors – Medical decisions are often influenced by the opinions and the decisions that have been taken by the physicians that already have examined the same patient.

As a consequence the probability of a serious error occurrence could be high and the probability of its recognition and correction very low. This frequently causes a repetition of exams in the same time or in different medical units and it slows down the diagnostic process (resources waste) and the proper treatment. So, proper actions for improving the working procedures have to be taken.

Correct medical information management and transmission is a key point [5], hence the introduction of telepathology can be relevant. There are several diagnostic discrepancies due to sampling error; those are a serious problem in telepathology. The difficulty of image standardization in telepathology is that so many factors can influence image quality [6,7]. The discussion about medical image standards commonly includes the discussion of required image resolution, the number of colours, monitor resolution, compression ratio, format etc. Pathology imaging has a wide range of requirements, and is subject to significant human factors and non-imaging-related parameters that make a single standard for pathology imaging problematic. Defining a required "pixel resolution" is meaningless if the optical focus or staining quality is not defined, and even if these parameters could be defined, the image type and quality required for some aspects of pathology is radically different from that required for others. One could decide on a file format for file transfer, but this would not address some of the more basic issues in pathology imaging. The important concepts for pathology imaging standards to consider are:

1. Systems should be able to share image files.

2. The standards should allow the transmission of information on baseline colours and recommended display parameters.

3. The images should be useful to the pathologist, not necessarily better or worse than direct examination of a slide under the microscope.

4. A mechanism to evaluate image quality objectively should be present.

5. A mechanism to adjust and correct minor errors of tissue processing should be developed.

6. A public organization should support pathologists in the development of standards.

To move pathology imaging into a space where standards can be effectively applied there are two main areas of attack. One involves formal training of pathologists in imaging and image-related activities. The second area is the development of technical mechanisms to remove human factor issues in the image capture process. The goals are to correct (or at least identify) differences between systems and materials, to develop technical protocols for evaluating and/or grading image quality objectively, and finally, to deploy colour standardization technology.

Image quality evaluation is another important factor. Currently, the methods used to evaluate image quality are very subjective and vary between individuals and institutions. This subjectivity does not necessarily affect diagnosis by telepathology and/or the imaging system [7]. However, as telepathology systems become more popular and more ubiquitous, it will become necessary to develop more objective image quality assessment methods. Even something as new and untested as the whole-slide imager is making rapid inroads into telepathology, and several commercial systems are available on the market. Each system gives a different visual impression of the images, and different models of the same system give different impressions and image quality. We do not know what level of quality of image we really need for clinical uses, for education or for research purposes, complicating the problem of standardization. The health care systems, and the education of health care personnel, have to be re-organized to systems that function in a cross-border fashion [8]. Prerequisites for this development shall be a specific emphasis on equity of access, interoperability and standardization of systems and protocols, security and legal aspects. There are technical, legal, organizational, and financial problems to be solved [9].

### Perspectives

The most important and perspective application of telepathology is education of health care professionals at a distance, so called distance education (eLearning). It has the abilities to apply new concepts, and ideas in which the learner becomes an owner of that knowledge, without any respect to distance. As such, telemedicine overall, and in particular telepathology education, is significantly part of health care revolution, since the event of modern medicine. Telepathology education process as a culture uses for the most part, distance education as the medium of dissemination of advanced information, and while it is an important aspect of today's education process, this medium should not be distracting, and the principles of learning and education should be unchanged. The addition of technology should not substitute for failed pedagogical process, but technology should allow that educational process, and the message to be disseminated, and tailored to individual groups and professionals, by retraining along some of the educational principles of traditional education.

While distance learning benefits are not challenged by most, it is difficult to estimate the impact on education overall. It should be measured by the content of the curriculum, which should be based on the process, perception, product and the mode of delivery. As such distance learning should be scrutinized just as traditional curriculum has been in the past and continue to be so. The only "change" should really be the medium of dissemination. Not the content per se, not the overall approach, and certainly not the end product, which is the education of the students, health professional and, the patients themselves. The differences between classical teaching and learning, and the new and modern form of teaching as well as learning is substantial in this new era. Instead of confined classroom teaching and learning, the entire universe has become a workplace, a learning environment, anywhere, anytime, 24 hours a day. This creates a sense of shared knowledge and virtual networking alliances. The demand for distance learning stems from the common sense of its applicability, but it requires the same standards of production, and evaluation of such programs. The main reasons to implement distance learning in health education are:

• Health care professional, in the information age, will acquire new skills and new knowledge without major disruption of their work.

• The need to reduce the cost of obtaining such education on new information (travel expenses, lodging, registration fees on venues like clinical conferences, congresses, and other forms of meetings).

• Need for better convergence of information age health care professional on communication and computing technologies.

Telemedicine in pathology training could supplement greatly pathology education of pathologists in countries with middle and low incomes without the expenses of moving those pathologists from one country to the other for supplemental education. Eventually, pathology education could be advanced to pathology telementoring, which could assist in the provision of pathology care to underserved areas and potentially facilitate the teaching of advanced pathology skills worldwide. Telepathology education is a very important element of overall progress in the telepathology. In order to be able to advance this, as an accepted culture and part of the daily practice of health care professionals, there are many initiatives that need to be taken, or an existing one to be supported.

## Conclusion

Perspectives and strategies for telepathology are currently evolving; as emerging operative requirements would allow self-sustainable large scale exploitation while recent technological developments are available to support integrated and cost-effective solutions to such requirements. However, as far as we know few telepathology services have proceeded to large scale exploitation, even after successful technological demonstration phases. Main exploitation drawbacks, problems and deficiencies have been:

1. Partial solutions approach instead of integrated total approach to health care assistance needs.

2. Lack of economical drive and consequently no self-sustainability for large scale exploitation.

3. Insufficient H24 (24 hours/day 365 days/year) medical and social operators support.

4. Insufficient networking approach for medical operators and scientific/clinical structures.

Telemedicine and telepathology are the most important for ensuring safe medical care. It is well known, that the first contact with patients needing medical help is the contact with the local primary care health center. Second opinions from specialists are often required in primary care health centers. An efficient and appropriate strategy of medical care can be worked out at the initial steps of patient's contact with health care. Such an approach can avoid unnecessary hospitalization, and will be a substantial contribution to the reduction of health costs.

By comparison with the usual health services telemedicine introduces added value and a positive impact at social, economic and cultural levels. Therefore, telemedicine is beginning to have an important impact on many aspects of healthcare in non-NATO countries. When implemented well telemedicine may allow these countries to leapfrog over their developed neighbours in successful healthcare delivery.
